# Serum microRNAs as biomarkers for the diagnosis of papillary thyroid carcinoma: A meta-analysis

**DOI:** 10.17305/bjbms.2022.7343

**Published:** 2022-06-04

**Authors:** Yuping Chen, Bingtian Dong, Lichun Huang, Huibin Huang

**Affiliations:** 1 Department of Endocrinology, The Second Affiliated Hospital of Fujian Medical University, Quanzhou, Fujian, China; 2 Department of Ultrasound, The Second Affiliated Hospital of Fujian Medical University, Quanzhou, Fujian, China

**Keywords:** Circulating microRNAs, serum, diagnosis, meta-analysis, papillary thyroid carcinoma

## Abstract

Papillary thyroid carcinoma (PTC) is the most common form of thyroid cancer. Several studies have proposed serum microRNAs (miRNAs) as novel biomarkers for diagnosing PTC. In this study, we conducted a meta-analysis aiming to investigate the overall diagnostic accuracy of serum miRNAs in PTC detection. Three online databases including PubMed, EMBASE, and Cochrane Library were searched up to 1 May 2021. We systematically reviewed studies evaluating the value of serum miRNAs in diagnosing PTC, and then summarized the area under receiver operating characteristics curve (AUROC), sensitivity, specificity, and diagnostic odds ratio to assess the accuracy of serum miRNAs for the discrimination between patients with PTC and patients with benign thyroid nodules and healthy controls. We included 32 studies from 6 articles. Overall, there were 463 PTC patients, 334 patients with benign thyroid nodules, and 104 healthy controls. The results showed that the summary sensitivity and specificity were 76% (95% confidence interval [CI]: 68-83%) and 86% (95% CI: 80-91%), respectively, and that the summary AUROC was 0.89 (95% CI: 0.86-0.91), when serum miRNAs were used for discriminating between PTC patients and those with benign nodules. On the other hand, the summary sensitivity and specificity of serum miRNAs for discriminating between PTC patients and healthy controls were 82% (95% CI: 77-86%) and 84% (95% CI: 76-90%), respectively, and the summary AUROC was 0.89 (95% CI: 0.86-0.92). We found that serum miRNAs have good diagnostic performance for the discrimination between patients with PTC and patients with benign nodules and healthy controls, and thus have considerable potential as novel minimally invasive tools for detecting PTC.

## INTRODUCTION

Over the past three decades, the global incidence of thyroid cancer has increased markedly, ranking sixth among all malignant tumors [1,2]. Among the various pathological types of thyroid cancer, papillary thyroid carcinoma (PTC) is the most common [3]. In fact, it has been estimated that PTC is responsible for approximately 80-90% of all thyroid cancers [4]. It is important to emphasize that papillary structures can also be observed in benign or malignant thyroid diseases; however, the treatment options are varied [5]. Therefore, the diagnostic distinction between benign and malignant diseases is crucial in determining clinical management, which may spare patients from unnecessary surgery [6].

Fine needle aspiration biopsy (FNAB) is considered to be the most reliable method to evaluate thyroid nodules [6]. As a thyroid morphological examination, FNAB has a high sensitivity for the differentiation between benign and malignant thyroid nodules [5]. It is, however, an invasive procedure and associated with several potential complications [5,7]. Moreover, unsatisfactory or non-diagnostic samples and suspicious/undetermined findings are the limitations of FNAB [8]. Notably, in the clinical setting, as many as 15-30% of FNAB samples cannot be accurately identified as benign or malignant [9]. In recent years, many researchers have increasingly focused their interest on the molecular biomarkers of PTC with the aim of providing improved diagnostic accuracy and avoiding unnecessary surgical treatments [3].

MicroRNAs (miRNAs) are a class of endogenous non-coding RNA molecules that regulate transcription and translation, which have gained tremendous attention in research in the last two decades given their ability to regulate gene expression [5,10]. Through the induction of translational repression or silencing effects by complementary binding to target messenger RNAs (mRNAs), these molecules play an important role in the regulation of gene expression [11-13]. At present, numerous research studies have documented that miRNAs are involved in the occurrence and development of various tumors through multiple mechanisms [14]. The previous studies have measured miRNAs from formalin-fixed, paraffin-embedded tissue, and the results showed that miR-221 and miR-222 were more expressed in PTC than in benign thyroid nodules [15]. Tissue miRNA profiles may be useful in the differential diagnosis of benign and malignant thyroid nodules [16]. Nevertheless, invasive procedures are still required to attain tissue miRNA profiles.

In contrast, samples of blood are easily available, essentially noninvasive, and can be collected at lower cost, all of which make blood samples attractive to explore for potential biomarkers [16]. Circulating miRNA molecules are very stable in plasma and serum, and can also be stably detected in peripheral blood [3,17]. In a recent meta-analysis, Xu et al. [5] demonstrated that circulating miRNAs have good diagnostic values to detect thyroid cancer and can be considered as promising diagnostic tools in clinical practice. To date, several studies have been published focusing on serum miRNAs in the detection of PTC, but the conclusions are not all consistent.

Molecular biomarkers have exhibited promising results in cancer, which may improve the preoperative management of patients [18]. In this study, we conducted a meta-analysis in order to investigate the overall diagnostic accuracy of serum miRNAs for the diagnosis of PTC. Furthermore, we also identified potential sources of heterogeneity through meta-regression and subgroup analyses.

## MATERIALS AND METHODS

### Search strategy

The preferred reporting items for systematic reviews and meta-analyses (PRISMA) guidelines were followed for reporting this systemic review and meta-analysis [19]. In our study, PubMed, EMBASE, and the Cochrane Library databases were searched until 1 May 2021 using the following terms: “microRNA,” “miRNA,” “miR,” “serum,” “circulation,” “thyroid carcinoma,” “thyroid cancer,” and “papillary thyroid carcinoma”. In addition, we also examined the references of the identified articles for other relevant publications. All search results were managed using EndNote X9 software.

### Selection criteria

The articles were included if they met the following criteria: (1) the study assessed the diagnostic accuracy of circulating serum miRNAs for the detection of PTC; (2) the study used pathological examination as the reference standard; and (3) sufficient data from the study was reported to construct a 2 × 2 table for test performance, including true positives, false positives, false negatives, and true negatives. The exclusion criteria were: (1) articles were abstracts, reviews, letters, case reports, and comments; (2) duplicate publications; (3) lack of sufficient data; (4) simple descriptive studies without control groups; and (5) not in English.

### Data extraction and quality assessment

Two researchers (CYP and DBT) independently screened the literature and extracted the data according to the inclusion and exclusion criteria described above. We extracted primary data from the studies, including first author, publication year, region, study design, patients’ age, patients’ gender, method of serum miRNAs detection, the target miRNAs, and the expression of miRNAs. In addition, the performance indices of different detected miRNAs were also extracted from each included article, including cutoff values, sensitivity, specificity, and area under the receiver operating characteristic curve (AUROC) values. In the present meta-analysis, if an article studied more than one miRNAs tests, we considered each miRNA test to be an independent study. Using the Quality Assessment of Diagnostic Accuracy Studies (QUADAS) tool [20], the quality of the included studies was assessed by two of our researchers (CYP and DBT) independently; discrepancies were resolved by consensus between the three researchers (CYP, DBT, and HHB).

### Assessment of heterogeneity and publication bias

The heterogeneity of the results between serum miRNAs studies was evaluated using the Cochrane-Q test. The inconsistency index I^2^ was calculated and then used to qualify the amount of non-threshold heterogeneity when an *I*^2^ ≥ 50%, heterogeneity may be assumed. To explore the potential sources of heterogeneity, we then performed meta-regression analyses using the following covariates: (1) Region (China vs. Italy); (2) publication year (≥ 2017 [median of all included studies] vs. < 2017); (3) study design (prospective vs. retrospective); (4) sample size (≥ 150 [median of all included studies] vs. < 150); (5) miRNA profiling (single miRNAs vs. combination miRNAs); and (6) internal reference (miR-16 vs. cel-miR-39). Publication bias was assessed using the Deeks’ funnel plot.

### Statistical analysis

The data were extracted from the included studies and then used to construct a series of 2 × 2 tables (including true positives, false positives, false negatives, and true negatives). First, the summary sensitivity and specificity, the summary positive likelihood ratio (PLR), negative likelihood ratio (NLR), and the summary diagnostic odds ratio (DOR) were calculated to examine the diagnostic accuracy of circulating serum miRNAs in PTC detection. In addition, the data from different studies included in our analysis were simultaneously used to construct the summary AUROC curves. Statistical analysis was performed using Stata 15.0 (STATA, College Station, Texas, USA).

## RESULTS

### Literature search

**FIGURE 1:**
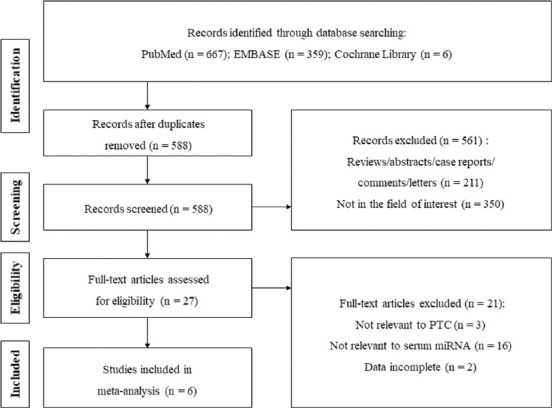
The study flow diagram. PTC: Papillary thyroid carcinoma; miRNA: microRNA.

Figure 1 displays the literature screening process. Through database searching, a total of 1032 articles were initially identified, including 667 from PubMed, 359 from EMBASE, and 6 from the Cochrane Library. After removal of 444 duplicates, a total of 588 studies were screened based on title and abstract. However, 582 studies were excluded for the following reasons: abstracts, reviews, not relevant to serum miRNAs diagnosis, non-serum samples, data incomplete, etc. Finally, six studies [16,21-25] were included for evaluation and meta-analysis.

### Characteristics of the included studies

The main characteristics of the included studies are summarized in Table 1. In total, 6 articles containing 32 studies were systematically reviewed. There were 463 PTC patients, 334 patients with benign thyroid nodules, and 104 healthy controls. All articles included in our meta-analysis were published between 2012 and 2018. Four of these articles were conducted in China, while two were from Italy. Four articles were prospective studies, and the remaining two retrospective. In addition, all articles included subjects from one center. For the detection of PTC, all articles used the results of pathological examination as the gold standard. There were three articles used benign thyroid nodule patients as controls, and the other three articles used both benign thyroid nodule patients and healthy people as controls. All articles performed reaction on serum samples, and reverse transcription quantitative polymerase chain reaction (RT-qPCR) was used for miRNA detection. Among the included studies, four articles used miR-16 as a reference for serum miRNA analysis, and two articles used cel-miR-39 as the reference. Five of the 32 studies analyzed combined miRNAs, and the remaining 27 studies analyzed single miRNAs.

**TABLE 1:**
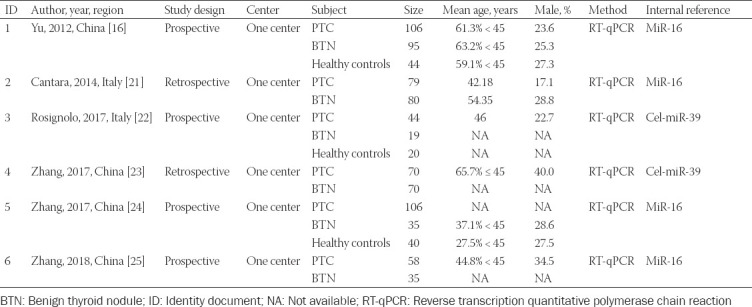
Characteristics of the studies included in this meta-analysis

### Diagnostic accuracy of serum miRNAs for detecting PTC

**FIGURE 2:**
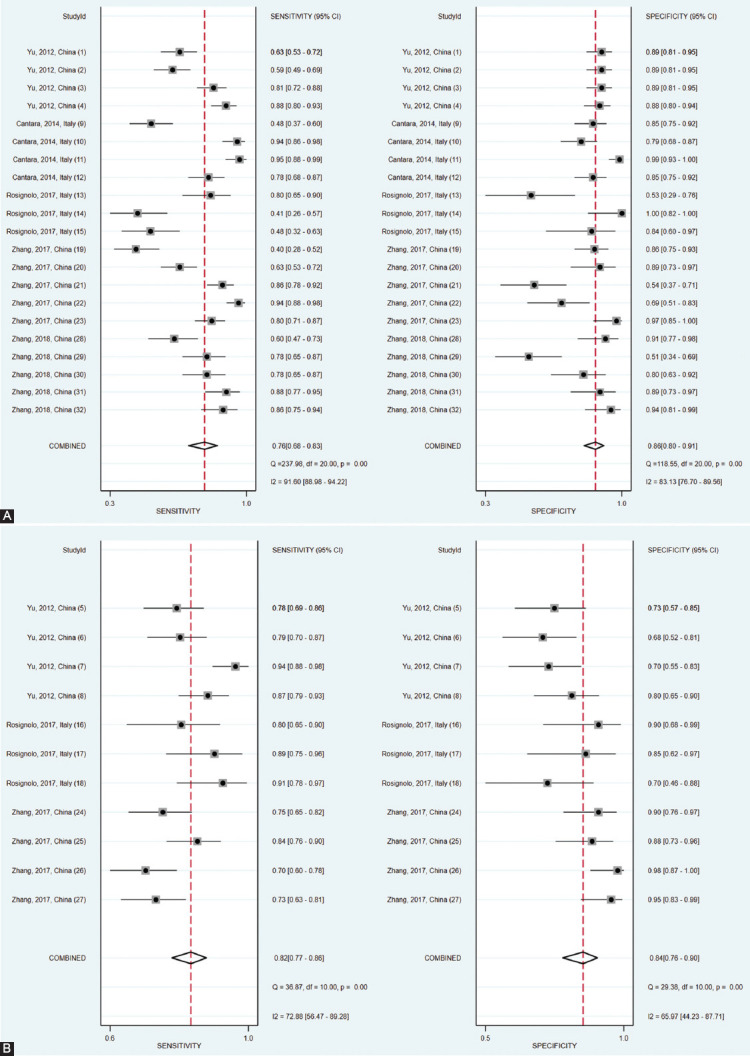
Coupled forest plots of the summary sensitivity and specificity of serum microRNAs for the discrimination of patients with papillary thyroid carcinoma from patients with benign nodules (A) and healthy controls (B).

A total of 21 studies investigated serum miRNAs for discriminating between PTC patients and those with benign thyroid nodules. Overall, 463 PTC patients and 334 patients with benign thyroid nodules were included for evaluation. Figure 2 shows that the summary sensitivity and specificity were 76% (95% confidence interval [CI]: 68-83%) and 86% (95% CI: 80-91%), respectively, when serum miRNAs were used for discriminating between PTC patients and those with benign thyroid nodules. Serum miRNAs diagnostic performance for discriminating between PTC patients and those with benign thyroid nodules is presented in Table 2, showing a mean AUROC value of 0.800 (range: 0.587-0.972). Results were then combined and we found that the summary AUROC value of serum miRNAs for discriminating between PTC patients and those with benign thyroid nodules was 0.89 (95% CI: 0.86-0.91) (Table 3). Moreover, the summary DOR and PLR of serum miRNAs were 20 (95% CI: 12-35) and 5.6 (95% CI: 3.8-8.0), respectively, for discriminating between PTC patients and those with benign thyroid nodules.

**TABLE 2:**
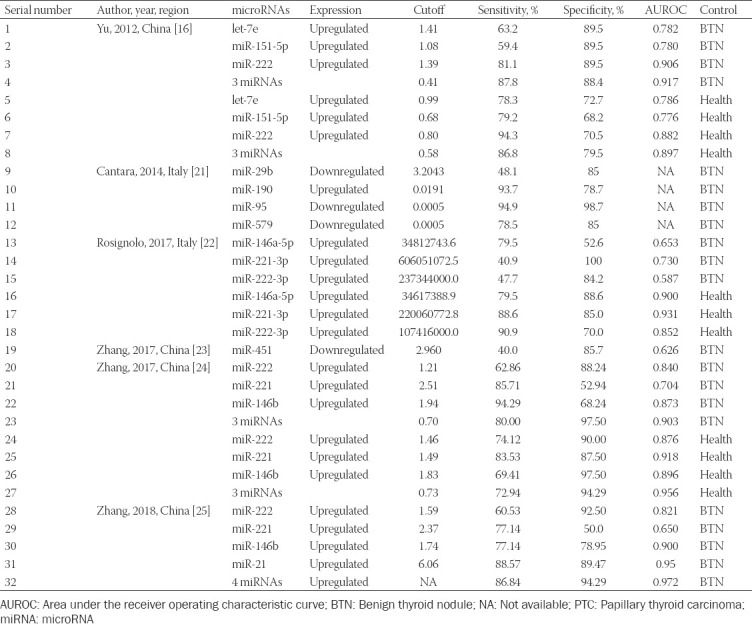
Summary of diagnostic accuracy of serum miRNAs for PTC

**TABLE 3:**
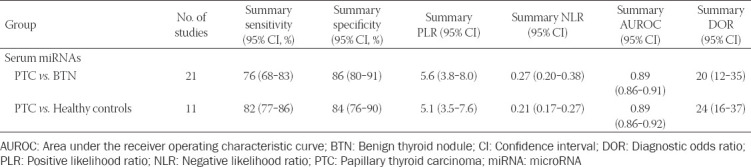
Meta-analysis results of serum miRNAs for PTC detection

**FIGURE 3:**
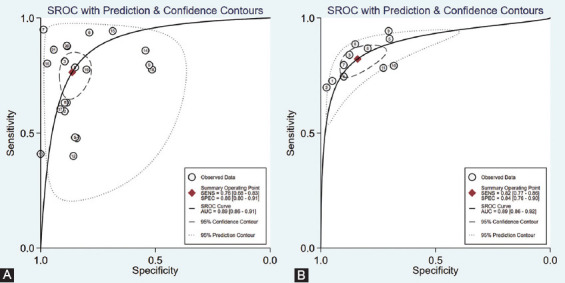
Summary receiver operating characteristic curves of the diagnostic performance of serum microRNAs for the discrimination of patients with papillary thyroid carcinoma from patients with benign nodules (A) and healthy controls (B).

On the other hand, 11 studies examined the performance of serum miRNAs in discriminating between PTC patients and healthy controls. A total of 463 PTC patients and 104 healthy controls were included for evaluation. As is shown in Table 3, the summary sensitivity and specificity of serum miRNAs for discriminating between PTC patients and healthy controls were 82% (95% CI: 77-86%) and 84% (95% CI: 76-90%), respectively. Table 2 shows that the mean AUROC value was 0.879 (range: 0.776-0.956). These results, when combined, reported that the summary AUROC value of serum miRNAs for discriminating between PTC patients and healthy controls was 0.89 (95% CI: 0.86-0.92) (Figure 3). Moreover, the summary DOR and PLR of serum miRNAs were 24 (95% CI: 16-37) and 5.1 (95% CI: 3.5-7.6), respectively, for discriminating between PTC patients and healthy controls.

Notably, several kinds of miRNAs, including miRNA-146b, miRNA-221, and miRNA-222, have been extensively studied. We examined the performance of serum miRNA-222 in discriminating between PTC patients and those with benign thyroid nodules, showing pooled sensitivity and specificity of 70% and 90%, respectively, and a summary AUROC of 0.946. In addition, the summary DOR and PLR of serum miRNA-222 were 22.55 (95% CI: 11.59-43.87) and 7.01 (95% CI: 4.44-11.06), respectively. Results are shown in Table 4.

**TABLE 4:**
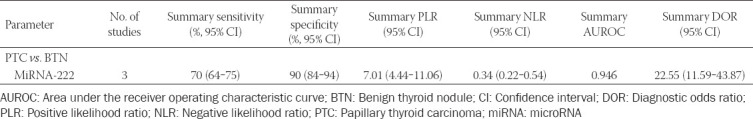
Meta-analysis results of serum miRNA-222 for PTC detection

### Heterogeneity and subgroup analysis

**FIGURE 4:**
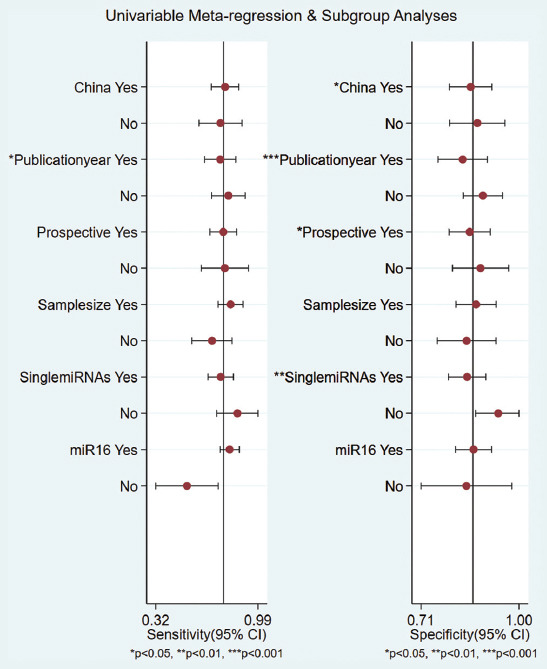
Meta-regression analysis of sensitivity and specificity of serum microRNAs for the discrimination of patients with papillary thyroid carcinoma from patients with benign nodules.

Substantial heterogeneity was observed in serum miRNAs studies (Figure 2). When serum miRNAs were used for discriminating between PTC patients and those with benign thyroid nodules, substantial heterogeneity was observed with regard to the summary sensitivity (*I*^2^ = 91.60%) and specificity (*I*^2^ = 83.13%). Meta-regression analysis was then conducted (Figure 4). We found that region, publication year, study design, and miRNA profiling could be the reasons of the observed heterogeneity. Other covariates, including sample size and internal reference, were not significant factors. To detect the factors that caused the heterogeneity, the subgroup analysis was performed. The results suggested that miR-16 as an internal reference was much better than cel-miR-39 in distinguishing PTCs from benign thyroid nodules, with a summary sensitivity of 80% vs. 52%, specificity of 86% vs. 82%, PLR of 5.9 vs. 3.0, NLR of 0.23 vs. 0.58, AUROC of 0.90 vs. 0.72, and DOR of 26 vs. 5. We also found that the studies published before 2017 have slightly higher accuracy than the later studies, and larger sample size studies have better diagnostic performance than smaller sample size studies in distinguishing PTCs from benign thyroid nodules. Of note, the combined miRNAs were significantly better than single miRNAs in distinguishing PTCs from benign thyroid nodules. Further, the subgroup analysis based on region and study design showed that the summarized diagnostic performances (sensitivity, specificity, PLR, NLR, AUROC, and DOR) were similar between China and Italy, and between prospective and retrospective studies. The detailed results on subgroup analysis are displayed in Table 5. Moreover, when serum miRNAs were used for discriminating between PTC patients and healthy controls, substantial heterogeneity was observed with regard to the summary sensitivity (*I*^2^ = 72.88%) and specificity (*I*^2^ = 65.97%).

**TABLE 5:**
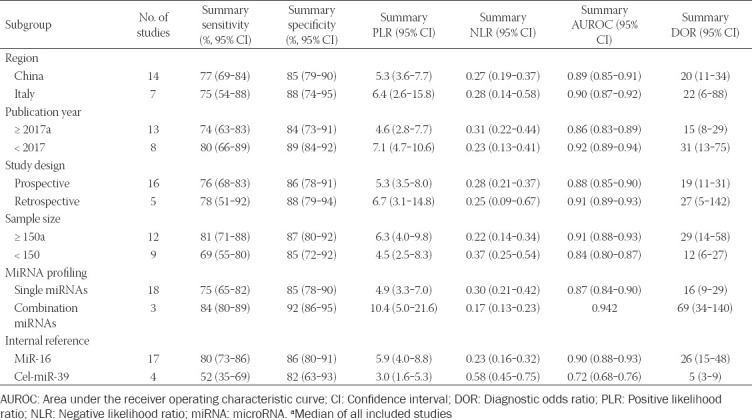
Summary of diagnostic criteria estimates and their 95% CI

### Publication bias

**FIGURE 5:**
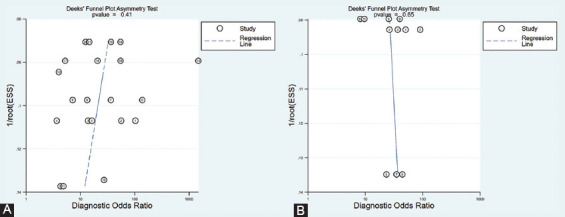
Deeks’ funnel plot asymmetry test for publication bias of serum microRNAs for the discrimination of patients with papillary thyroid carcinoma from patients with benign nodules (A) and healthy controls (B).

Figure 5 illustrates the Deeks’ funnel plots of serum miRNAs. The results showed that there was no publication bias for serum miRNAs for discriminating between PTC patients and those with benign thyroid nodules (*p* = 0.41). In addition, no evidence of publication bias was demonstrated for serum miRNAs for discriminating between PTC patients and healthy controls (*p* = 0.65).

## DISCUSSION

In this study, we conducted a meta-analysis with the aim to systematically review the current published data on the diagnostic accuracy of circulating serum miRNAs for PTC detection. We included 32 studies from 6 articles. Overall, a total of 463 PTC patients, 334 patients with benign thyroid nodules, and 104 healthy controls were included in the present meta-analysis for evaluation.

Our study revealed that circulating serum miRNAs have good diagnostic performance to diagnose PTC and distinguish PTC patients from patients with benign thyroid nodule. The results showed that the summary sensitivity and specificity were 76% (95% CI: 68-83%) and 86% (95% CI: 80-91%), respectively, and the summary AUROC value was 0.89 (95% CI: 0.86-0.91), when serum miRNAs were used for discriminating between PTC patients and those with benign thyroid nodules. Similarly, the summary sensitivity and specificity of serum miRNAs for discriminating between PTC patients and healthy controls were 82% (95% CI: 77-86%) and 84% (95% CI: 76-90%), respectively, and the summary AUROC value was 0.89 (95% CI: 0.86-0.92). Our findings thus indicated that serum miRNAs can be used as novel, promising, and minimally invasive diagnostic tools for detecting PTC in clinical practice.

The incidence of thyroid cancer has increased ­progressively worldwide [26]. In clinical practice, ruling out malignancy is the main goal of diagnostic workup for thyroid nodules [27]. It is estimated that as many as 50% of PTC surgeries are unnecessary [28]. FNAB is the most frequently used diagnostic method but is invasive with some disadvantages, such as undefined significance in describing lesions [29,30]. Hence, there is still a need to find a cost-effective and non-invasive method for the diagnosis of PTC. In recent years, great efforts have been devoted to identifying miRNA expression changes in different diseases and cancer types, with the aim of finding sensitive and objective biomarkers. As the disease progresses, specific miRNAs are released from the tissue into the circulation, which were highly stable both in plasma and in serum [31]. These facts encourage further studies on these non-invasive biomarker tests. In our study, we identified many serum miRNAs associated with the development of PTC. Several kinds of miRNAs, including miRNA-146b, miRNA-221, and miRNA-222, have been widely studied. Surprisingly, our results showed that serum miRNA-222 has high diagnostic accuracy for discriminating between PTC patients and those with benign thyroid nodules, with pooled sensitivity, specificity, AUROC, and DOR values of 70%, 90%, 0.946, and 22.55, respectively. MiRNA-222 is a well-defined proto-oncogene family, which was shown to be abnormally expressed in many tumors, such as primary hepatocellular carcinoma and pancreatic cancer [32]. Kondrotiene et al. [33], in a recent original study, examined the diagnostic value of plasma-derived miR-222 for differentiation of PTC from benign thyroid nodule and reported sensitivity and specificity of 61.2% and 78.3%, respectively, and an AUROC of 0.711, both of which were much lower than our results. This suggested that serum miR-222 may be more conducive in distinguishing PTCs from benign thyroid nodules than plasma miR-222. In this meta-analysis, however, we only included three articles about the relationship between miRNA-222 and PTC. Larger studies are therefore necessary to verify these results.

Similarly, Xu et al. [5] investigated the diagnostic accuracy of circulating miRNAs in thyroid cancer and a subgroup analysis in specimen (plasma vs. serum) was performed. The results of subgroup analysis in specimen showed that the summary specificity, PLR, AUROC, and DOR of serum miRNAs in distinguishing thyroid cancers from benign thyroid nodules were higher than those of plasma miRNAs, although the summary sensitivity was slightly lower than that of plasma miRNAs (79% vs. 84%).

There is an urgent need to develop novel and noninvasive markers for tumor detection, prediction, and prognosis for the management of cancer [34]. In this regard, miRNAs appear to be the most promising [34]. Several meta-analyses have been recently published, which studied miRNAs as molecular biomarkers in the diagnosis of thyroid cancer. Of interest, Xu et al. [5] found that the summary sensitivity, specificity, and AUROC of circulating miRNAs were 81%, 81%, and 0.88 for distinguishing thyroid cancers from benign thyroid nodules, respectively; and 81%, 85%, and 0.89 for distinguishing thyroid cancers from healthy controls, respectively. The results of that study were similar to ours. In summary, circulating miRNAs can be used as auxiliary tools for diagnosing malignancy in clinical practice and avoiding unnecessary surgeries [5].

Noticeably, substantial statistical heterogeneity was present in our meta-analysis. Thus, we conducted meta-regression and subgroup analyses in order to explore the confounding factors. The results demonstrated that region, publication year, study design, and miRNA profiling could be the reasons for the heterogeneity in the summary sensitivity and specificity of serum miRNAs for discriminating between PTC patients and those with benign thyroid nodules; however, other covariates including sample size and internal reference were not significant factors. It is noteworthy that the results of subgroup analysis in terms of number of miRNAs showed that the combined miRNAs were better than single miRNAs in distinguishing PTCs from benign thyroid nodules, with a summary sensitivity of 84% vs 75%, specificity of 92% vs. 85%, AUROC of 0.94 vs. 0.87, and DOR of 69 vs. 16. The findings are in agreement with previous study focusing on thyroid cancer detection by Xu et al. [5]. As a result, future research could pay more attention to the combined detection of miRNAs to provide improved accuracy.

We acknowledge that some limitations still exist in our meta-analysis. First, we only focused on the full articles published in English, which may bias the results to some extent. Second, the number of articles on circulating serum miRNAs for the diagnosis of PTC was not adequate, so we could not set more stringent inclusion criteria. Furthermore, all included studies were conducted in China and Italy, which limited our conclusions for other ethnic populations. Future large prospective studies will, therefore, be needed. Third, cutoff values of serum miRNAs varied across the included studies because of the heterogeneous internal references and the different distribution stages of PTC in the experimental group, which can cause heterogeneity. Finally, our study may have several inherent limitations, such as the inability to identify optimal thresholds for serum miRNAs to detect PTC and the heterogeneity that may exist.

## CONCLUSION

Our results demonstrated that serum miRNAs have good diagnostic performance for the discrimination between patients with PTC from patients with benign thyroid nodules and healthy controls. Serum miRNAs can, therefore, be considered as novel, promising, and minimally invasive diagnostic tools for PTC in clinical practice. Additional studies with larger patient samples focusing on combination miRNAs will be needed to further validate their accuracy in PTC detection.
